# Upregulation of UCP2 by Adiponectin: The Involvement of Mitochondrial Superoxide and hnRNP K

**DOI:** 10.1371/journal.pone.0032349

**Published:** 2012-02-16

**Authors:** Mingyan Zhou, Aimin Xu, Paul K. H. Tam, Karen S. L. Lam, Bosheng Huang, Yan Liang, In-Kyu Lee, Donghai Wu, Yu Wang

**Affiliations:** 1 Department of Pharmacology and Pharmacy, University of Hong Kong, Hong Kong, China; 2 Department of Medicine, University of Hong Kong, Hong Kong, China; 3 Department of Surgery, University of Hong Kong, Hong Kong, China; 4 School of Medicine, Kyungpook National University, Daegu, Korea; 5 Key Laboratory of Regenerative Biology, Guangzhou Institute of Biomedicine and Health, Chinese Academy of Sciences, Guangzhou, China; The University of Hong Kong, Hong Kong

## Abstract

**Background:**

The adipocyte-derived hormone adiponectin elicits protective functions against fatty liver diseases and hepatic injuries at least in part by stimulating the expression of a mitochondrial inner membrane transporter, uncoupling protein 2 (UCP2). The present study was designed to investigate the cellular and molecular mechanisms underlying adiponectin-induced UCP2 expression.

**Methodology/Principal Findnigs:**

Mice were treated with adiponectin and/or different drug inhibitors. Parenchymal (PCs) and nonparenchymal (NPCs) cells were fractionated from the liver tissues for mitochondria isolation, Western blotting and quantitative PCR analysis. Mitochondrial superoxide production was monitored by MitoSOX staining and flow cytometry analysis. Compared to control mice, the expression of UCP2 was significantly lower in NPCs, but not PCs of adiponectin knockout mice (AKO). Both chronic and acute treatment with adiponectin selectively increased the mRNA and protein abundance of UCP2 in NPCs, especially in the enriched endothelial cell fractions. The transcription inhibitor actinomycin D could not block adiponectin-induced UCP2 expression, whereas the protein synthesis inhibitor cycloheximide inhibited the elevation of UCP2 protein but not its mRNA levels. Mitochondrial content of heterogeneous nuclear ribonucleoprotein K (hnRNP K), a nucleic acid binding protein involved in regulating mRNA transportation and stabilization, was significantly enhanced by adiponectin, which also evoked a transient elevation of mitochondrial superoxide. Rotenone, an inhibitor of mitochondrial respiratory complex I, abolished adiponectin-induced superoxide production, hnRNP K recruitment and UCP2 expression.

**Conclusions/Significance:**

Mitochondrial superoxide production stimulated by adiponectin serves as a trigger to initiate the translocation of hnRNP K, which in turn promotes UCP2 expressions in liver.

## Introduction

Non-alcoholic fatty liver disease (NAFLD) is one of the metabolic syndrome components closely associated with obesity, a worldwide pandemic [1]. The presence of steatosis in liver poses significant risks for the development of Type 2 Diabetes, cardiovascular diseases, viral hepatitis, drug-induced hepatotoxicity and alcoholic steatohepatitis [2], [3], [4]. In western countries, NAFLD is the most frequent hepatic lesion with an estimated prevalence of 10–25% [5]. About 20% to 30% of individuals with NAFLD progress into non-alcoholic steatohepatitis (NASH), cirrhosis and hepatocellular carcinoma [6], [7].

Adiponectin is an adipocyte-derived hormone possessing a wide range of beneficial functions against obesity-associated medical complications [8], [9], [10]. The hepatoprotective activities of adiponectin have been demonstrated by evidence derived from clinical, genetic and pharmacological studies [11], [12], [13], [14], [15], [16], [17], [18]. Epidemiological investigations suggest that low adiponectin level is an independent risk factor for NAFLD and liver dysfunctions in different ethnic groups [11], [12], [15], [17], [18], [19], [20]. In mice, adiponectin deficiency leads to exacerbated liver injuries induced by chemicals, endotoxins, alcohol consumption and obesity [21], [22], [23], [24], whereas administration of this protein protects against fatty liver diseases, as well as various other forms of hepatic injuries [17], [25], [26], [27], [28]. In adiponectin knockout (AKO) mice, there is a pre-existing condition of hepatic steatosis and mitochondria dysfunction, characterized by abnormal ultrastructures and defective mitochondrial respiratory chain (MRC) activity [24]. Adiponectin treatment restores mitochondrial functions, depletes lipid accumulation, and up-regulates the mRNA and protein expression of uncoupling protein 2 (UCP2) in liver tissues of AKO mice. UCP2 is a mitochondrial ion carrier encoded by nuclear genome but functions exclusively in mitochondria [29]. Although the detailed physiological functions of UCP2 remain to be elucidated, it has been suggested that increased expression of UCP2 may help to prevent the development of hepatic steatosis and steatohepatitis [30]. The liver protective functions of adiponectin are significantly attenuated in UCP2 knockout mice [24]. Administration with adiponectin or UCP2 produces similar effects on MRC activity, fatty acyl CoA accumulation, oxidative stress and inflammation in the liver tissues of AKO mice [31]. These information suggest that upregulation of UCP2 plays an essential role in mediating the hepatoprotective functions of adiponectin. On the other hand, the underlying cellular and molecular mechanisms by which adiponectin stimulate UCP2 expression in liver are largely unknown. Results in the present study demonstrate that adiponectin promotes UCP2 expression selectively in nonparenchymal cells, especially in hepatic endothelial cells, by provoking mitochondrial superoxide production, which facilitates the transportation, stabilization and translation of UCP2 mRNA.

## Results

### Adiponectin treatment enhanced UCP2 expressions in nonparenchymal cells

**Figure 1:**
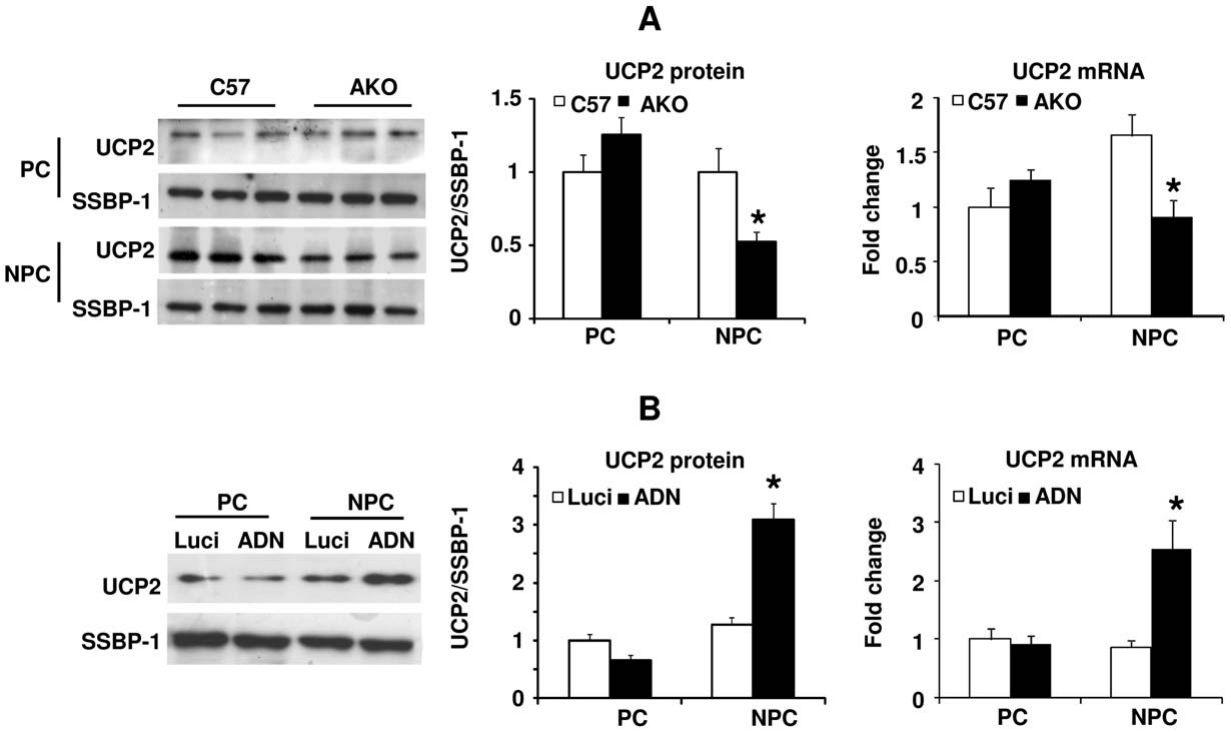
UCP2 expression is decreased in NPCs of the AKO mice liver. *A*, PCs and NPCs were isolated from the liver tissues of C57 and AKO mice. The protein (left and middle panel) and mRNA (right panel) expression of UCP2 was measured by Western blotting and QPCR, respectively. SSBP-1 was used as the protein loading control and β-actin as the internal control for quantifying gene expressions. QPCR results were plotted as fold changes against the C57 PC samples. *, *P*<0.05 vs C57 NPC samples, n = 3. *B*, AKO mice were treated with adenoviruses encoding luciferase (Luci) or adiponectin (ADN). UCP2 protein (left and middle panel) and mRNA (right panel) levels in PCs and NPCs were analyzed as above. QPCR results were presented as fold changes against the Luci PC controls. *, *P*<0.05 vs corresponding controls, n = 3.

**Figure 2:**
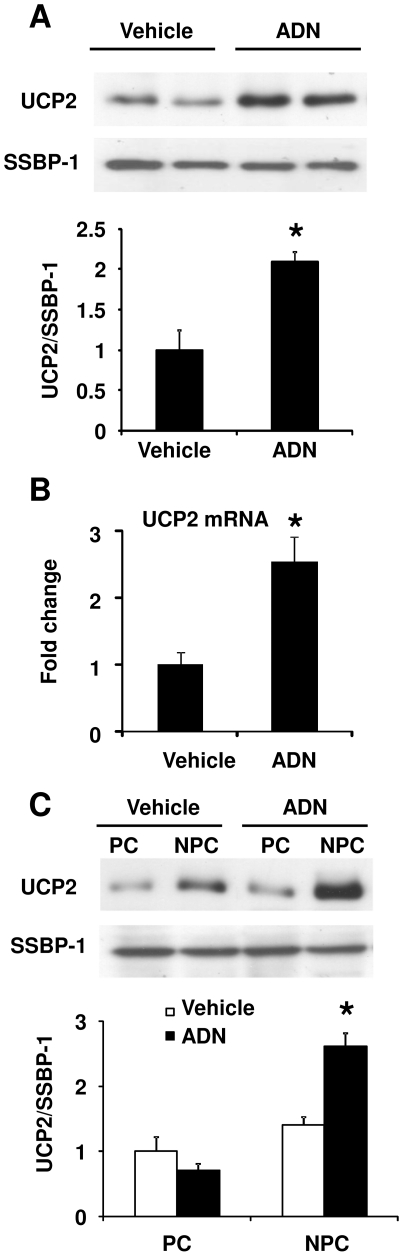
Acute treatment with adiponectin elevates UCP2 expression in NPCs. PBS or 50 µg of murine adiponectin protein was injected into the portal vein of AKO mice livers. Liver tissues were collected for evaluation of UCP2 expression. The protein abundance in mitochondria (A), mRNA expression in total tissue lysates (B), and the protein content in PC and NPC fractions (C) were analyzed as in Figure 1. *, *P*<0.05 vs vehicle treated samples, n = 3.

To determine the effect of adiponectin on UCP2 expression in parenchymal (PCs) and nonparenchymal (NPCs) cells, Western blotting and quantitative RT-PCR (QPCR) were performed on cells isolated from the livers of C57 and AKO mice. The protein and mRNA abundance of UCP2 in PCs isolated from AKO mice was not different from that in C57 mice (Figure 1A). The UCP2 protein abundance was lower in NPCs isolated from AKO mice compared to that in C57 mice (Figure 1A, left and middle panel). The mRNA level of UCP2 in NPCs isolated from AKO mice was ∼50% of the C57 mice (Figure 1A, right panel). Administration of adenovirus encoding adiponectin increased the protein and mRNA abundance of UCP2 in NPCs, but not PCs, of AKO mice (Figure 1B). Next, the effect of acute recombinant protein treatment on UCP2 expression was evaluated. Both mitochondrial protein abundance and the mRNA level of UCP2 was significantly up-regulated at 30 minutes following administration of the protein into portal vein of AKO mice liver (Figure 2, A and B). Similarly, the elevated UCP2 expression was mainly found in NPCs (Figure 2C).

### UCP2 was induced by adiponectin treatment in hepatic endothelial cells

**Figure 3:**
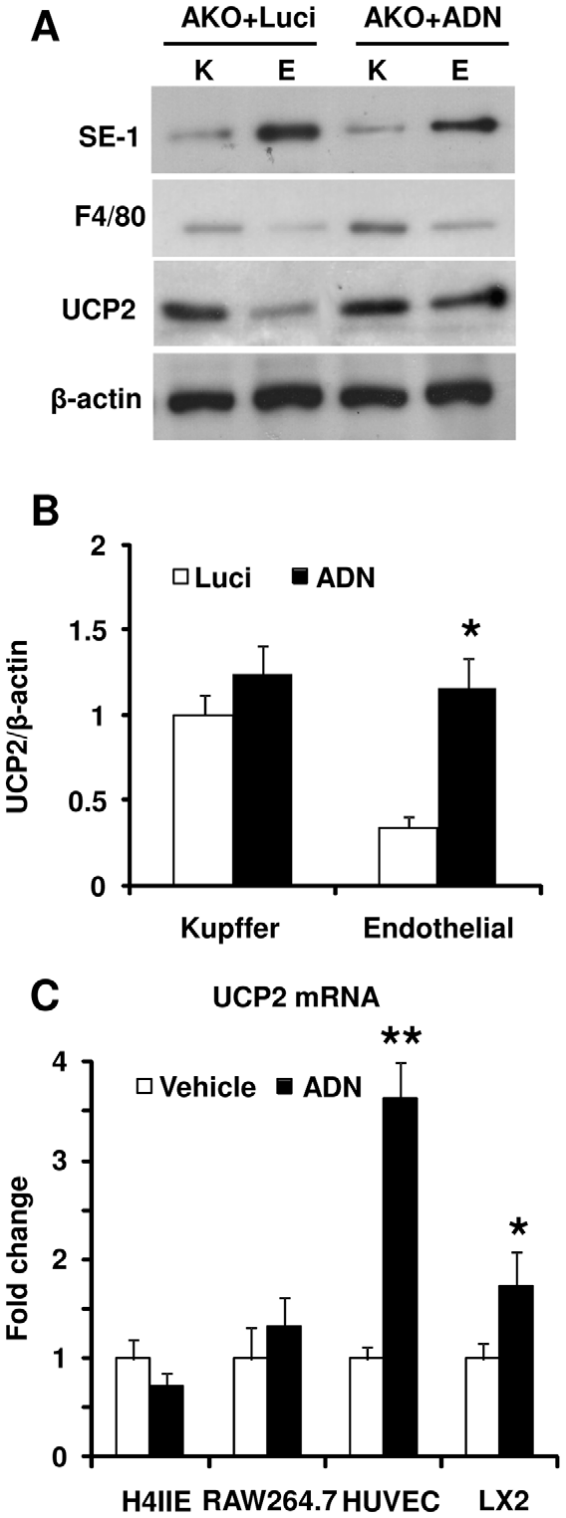
Adiponectin promotes UCP2 expression in hepatic endothelial cells. AKO mice were treated as in Figure 1B. The NPCs were used for further fractionation to collect those enriched with Kupffer (K)- and sinusoidal endothelial (E) cells. The enrichment of the two cell types were confirmed by Western blotting using macrophage marker F4/80 and sinusoidal endothelial marker SE-1, respectively (A). UCP2 expression was monitored as in Figure 1. After densitometry analysis, the protein ratio of UCP2/β-actin was calculated and presented as fold changes against Luci Kupffer samples (B). UCP2 gene expression was also quantified in four types of cells treated with or without adiponectin (10 µg/ml) (C). *, *P*<0.05 and **, *P*<0.01 vs corresponding controls, n = 3.

Liver consists of parenchyml (PC) and nonparenchymal (NPC) cells. Hepatocytes are the components of PCs and other cell types, including Kupffer cells (∼30%), endothelial cells (∼60%), stellate cells, and leukocytes etc, are grouped in NPCs. To further determine the cellular target of adiponectin, enriched fractions containing Kupffer cells and endothelial cells were used for Western blotting to determine the mitochondrial UCP2 content. The efficiency of cellular enrichment was validated by Western blotting using macrophage marker F4/80 and endothelial cell marker SE-1 (Figure 3A). Compared to that of Kupffer cells, the protein abundance of UCP2 was much lower in endothelial cells. While adiponectin treatment had no significant effect on UCP2 expression in Kupffer cells, it markedly increased the UCP2 content (by approximately 3-fold) in the endothelial fractions harvested from AKO mice liver (Figure 3, A and B). To further corroborate the cell type-specific effect of adiponectin, human umbilical vein endothelial cells (HUVEC), hepatoma H4IIE, macrophage RAW 264.7 and stellate LX-2 cell lines were treated with or without adiponectin. QPCR analysis revealed that adiponectin significantly increased UCP2 mRNA level by ∼3.5- and ∼1.8-fold in HUVEC and LX-2, respectively (Figure 3C). Consistent with the *in vivo* data, the UCP2 expression in hepatoma cell H4IIE and macrophage cell RAW 264.7 was not affected by adiponectin (Figure 3C).

### Adiponectin enhanced the mRNA stability of UCP2 and promoted hnRNP K translocation to mitochondria

**Figure 4:**
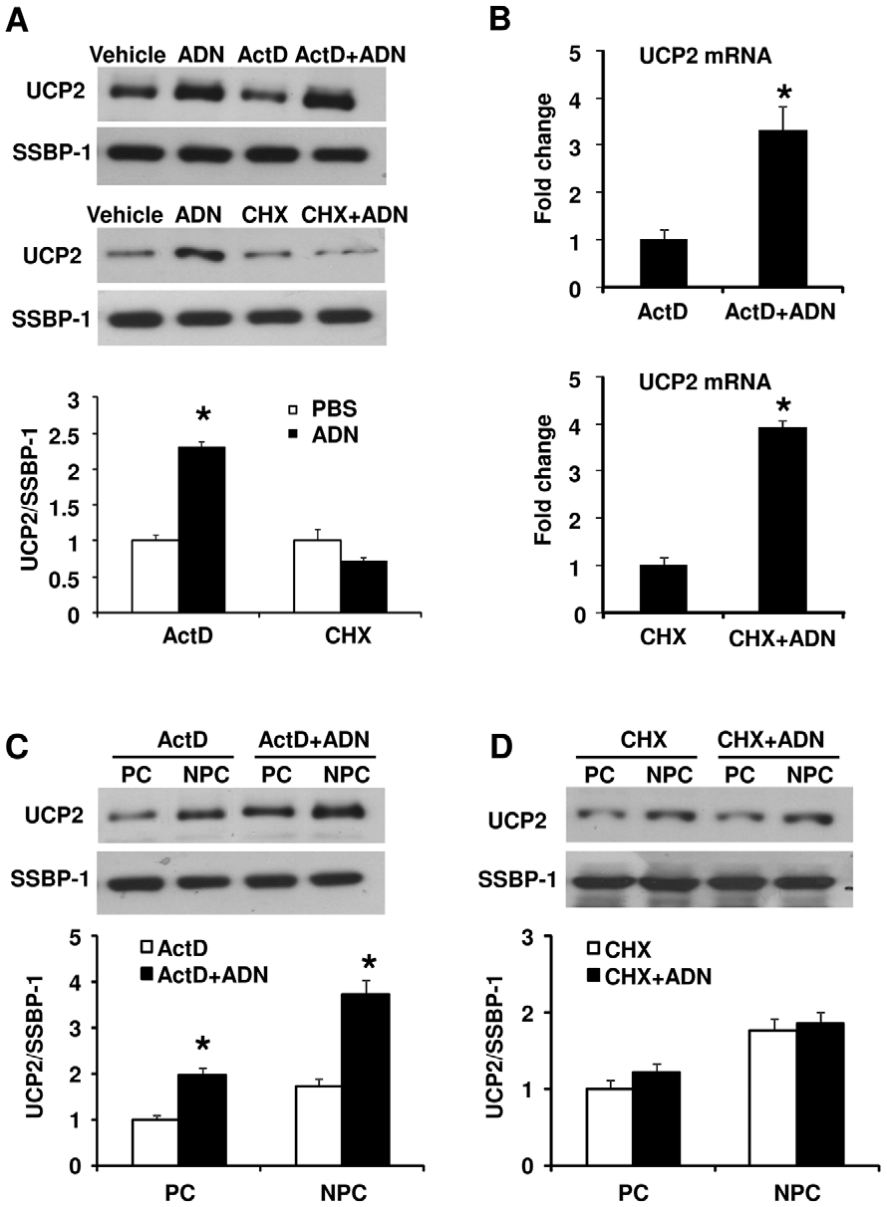
Adiponectin enhances the mRNA stability of UCP2 and promotes its protein synthesis. Two inhibitors, actinomycin D (ActD, A, B and C) or cycloheximide (CHX, A, B and D), were administered together with or without adiponectin protein into liver tissues of AKO mice livers. The protein (A) and mRNA (B) abundance of UCP2 was monitored by Western blotting and QPCR, respectively. The relative protein expression was also monitored in PCs/NPCs lysates (C and D). *, *P*<0.05 vs corresponding controls, n = 3.

To investigate the mechanism underlying adiponectin-induced UCP2 expression, PBS or adiponectin was administered into the portal vein of AKO mice in the presence or absence of transcriptional inhibitor actinomycin D (ActD) or protein synthesis inhibitor cycloheximide (CHX) (Figure 4). ActD treatment did not block the effects of adiponectin on UCP2 expression. In the presence of ActD, adiponectin increased the UCP2 protein and mRNA levels by ∼2.3- and ∼3.2-fold, respectively (Figure 4, A and B). The elevation of UCP2 protein expression was observed in both PC and NPC fractions. On the other hand, CHX completely suppressed the stimulatory effect of adiponectin on UCP2 protein expression, which was measured in the lysates derived from both the AKO liver tissues and the NPC fractions (Figure 4, A and D). However, CHX did not prevent adiponectin-induced elevation of UCP2 mRNA expression (Figure 4B).

**Figure 5:**
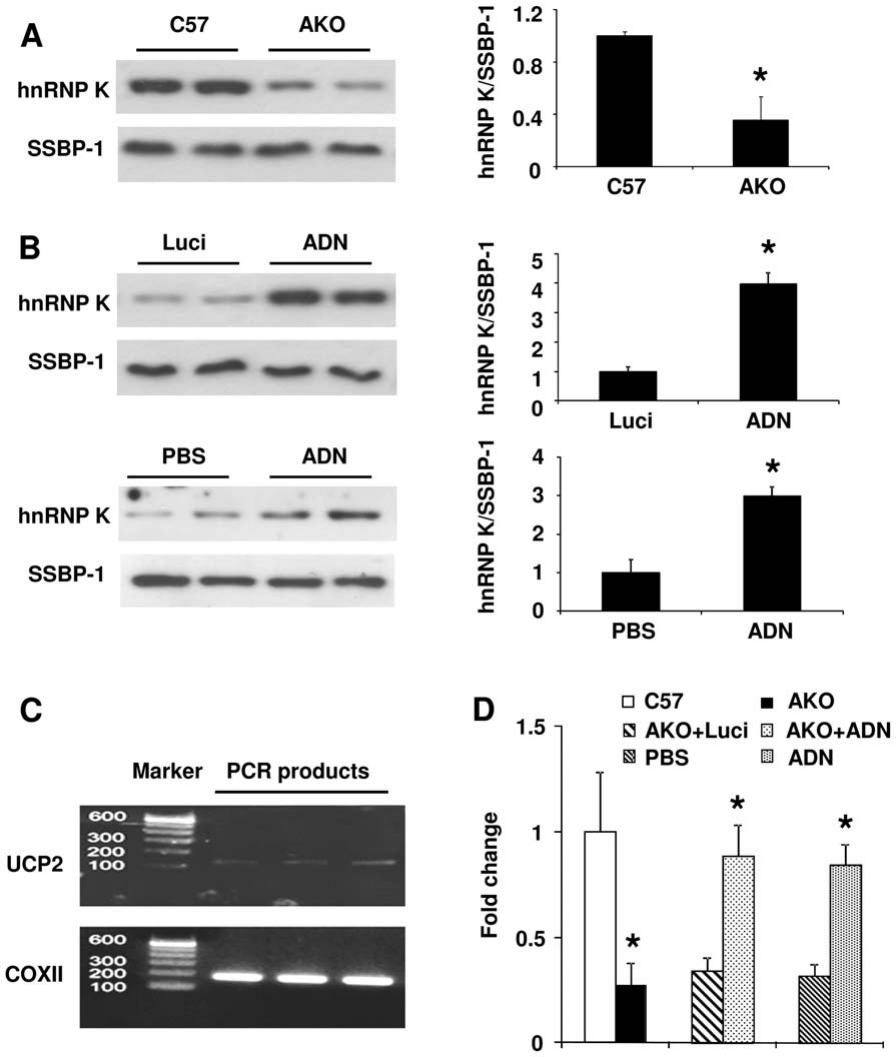
Mitochondrial content of hnRNP K protein is decreased in AKO mice. Mitochondria were isolated from C57 mice and AKO mice (A), or AKO mice subjected to chronic or acute treatment with adiponectin (B). hnRNP K protein content in mitochondrial fractions was measured by Western blotting (A and B). RNA was extracted from the mitochondrial fractions and used for QPCR analysis. The PCR products were visualized in agarose gel for confirming the specific products (C). The QPCR results were compared and presented as fold changes against C57 samples (D). The gene encoding COXII was used as the internal control. *, *P*<0.05 vs corresponding controls, n = 3.

Heterogeneous nuclear ribonucleoprotein K (hnRNP K) is involved in regulating UCP2 mRNA stability and protein synthesis [32]. While the total hnRNP K protein expression was not significantly different between C57 and AKO mice (data not shown), the abundance of this protein was much lower in the mitochondria fractions of AKO mice liver compared to those of C57 mice (Figure 5A). Adenovirus-mediated chronic over-expression of adiponectin significantly elevated hnRNP K levels in mitochondria isolated from AKO mice (Figure 5B, top panel). Similarly, injection of recombinant adiponectin, via portal vein, also increased the mitochondrial hnRNP K protein content (Figure 5B, bottom panel). UCP2 mRNA is transported to mitochondria-associated polysomes for protein synthesis. A 123 bp specific fragment of UCP2 mRNA was amplified from mitochondrial RNA samples (Figure 5C). The mitochondrial contents of UCP2 mRNA were quantified by QPCR using the mitochondrial gene cytochrome C oxidase subunit II (COX II) as the internal control (Figure 5D). It was found that UCP2 mRNA content in the mitochondria-associated polysome fraction of AKO mice was only ∼29.1% of that in the C57 group. Adiponectin replacement by either adenovirus administration or portal vein injection restored the UCP2 mRNA contents (Figure 5D).

### Treatment with rotenone, a MRC complex I inhibitor, abolished adiponectin-mediated induction of UCP2 and hnRNP K translocation

**Figure 6:**
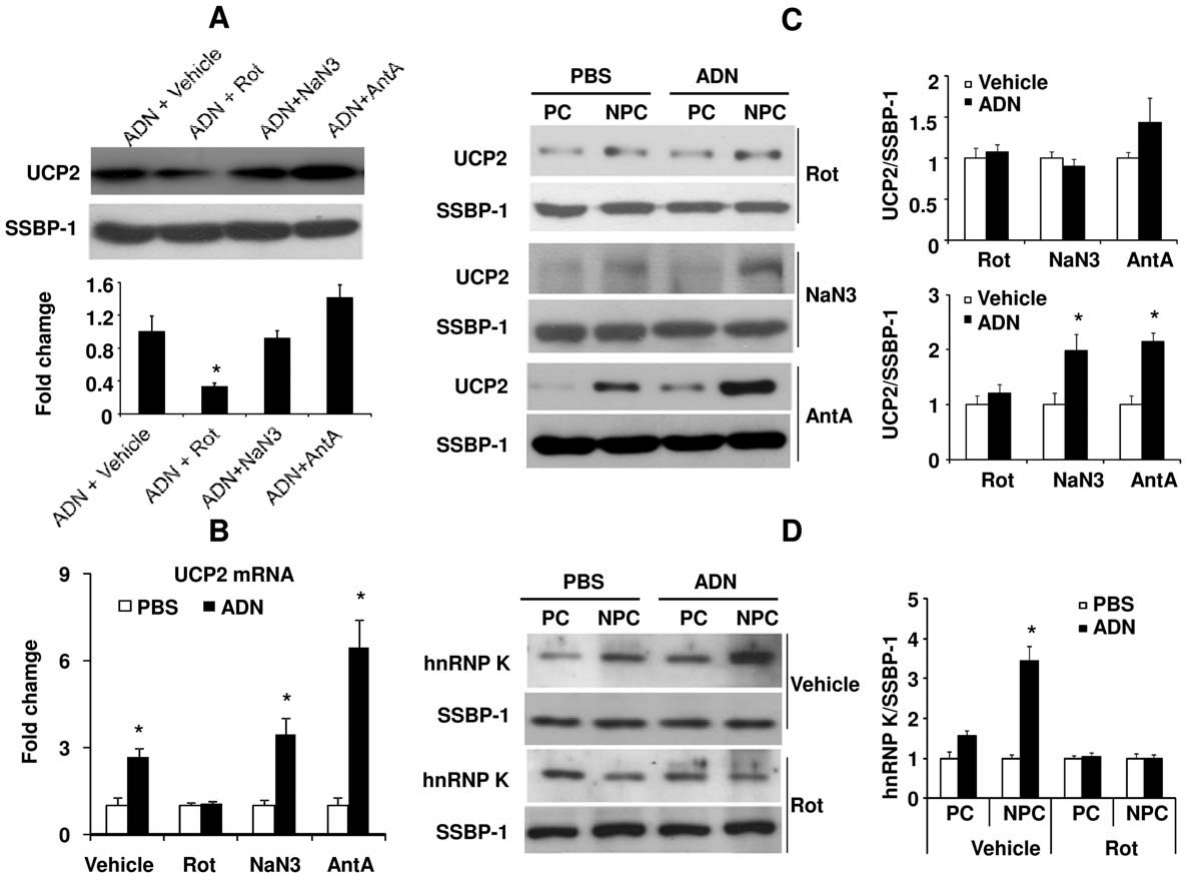
Rotenone inhibits adiponectin-stimulated UCP2 expression and hnRNP K translocation to mitochondria. Mitochondrial respiration chain inhibitors, including rotenone (Rot), sodium azide (NaN_3_) and antimycin A (AntA) were administered into liver tissues of AKO mice as described in Methods. The effects of each inhibitor on adiponectin-induced UCP2 protein expression in mitochondria of liver tissues (A), UCP2 mRNA expression in total tissue lysates (B) were evaluated. The mitochondria protein content of UCP2 (C) and hnRNPK (D) in PCs and NPCs were compared by Western blotting. *, *P*<0.05 vs corresponding controls, n = 3.

Western blotting was performed on mitochondria protein lysates of AKO mice treated with recombinant adiponectin in the presence or absence of of MRC complexes inhibitors, including rotenone (Rot, complex I inhibitor), antimycin A (AntA, complex III inhibitor) and sodium azide (NaN_3_, complex IV inhibitor). The stimulatory effect of adiponectin on mitochondrial UCP2 protein expression was significantly attenuated by rotenone, but not by antimycin A or NaN_3_ (Figure 6A). Adiponectin-induced elevation of UCP2 mRNA level was also attenuated by rotenone treatment (Figure 6B). This inhibitor mainly blocked the expression of UCP2 in NPCs (Figure 6C). Rotenone treatment also prevented adiponectin-induced mitochondria translocation of hnRNP K in NPCs (Figure 6D).

**Figure 7:**
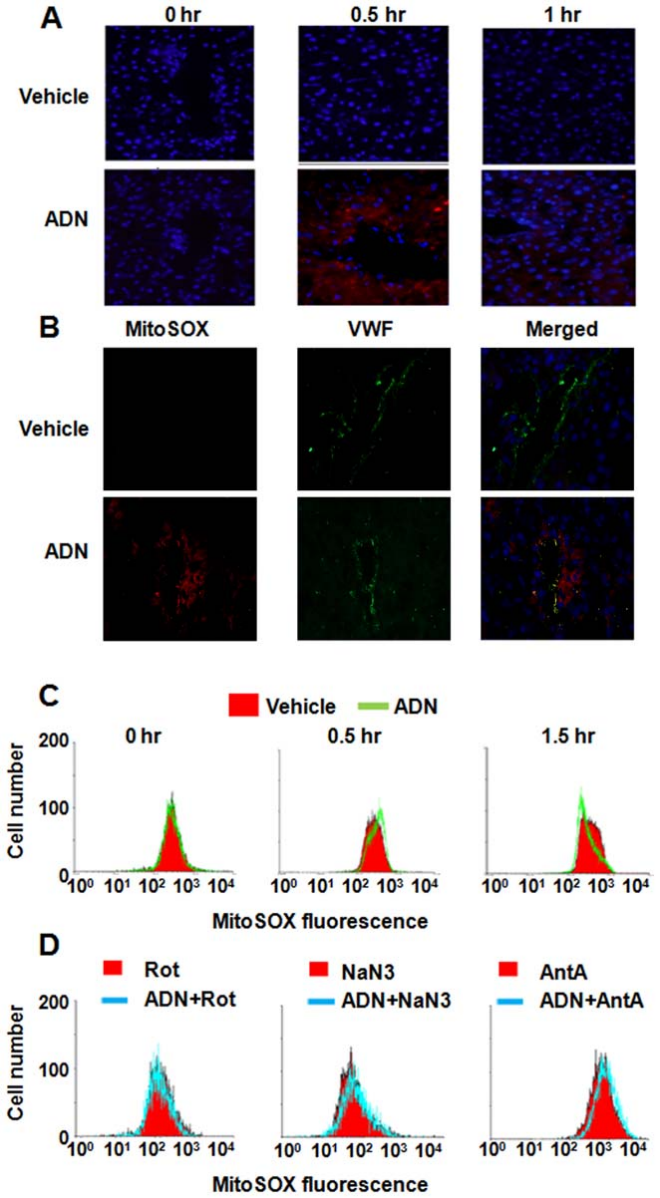
Adiponectin treatment induces transiently elevates mitochondrial superoxide. Liver sections (A and B) and HUVEC samples (C) were prepared before and at different time points after vehicle (PBS) or adiponectin treatment. MitoSOX and VWF staining was performed as described in Methods. Note that rotenone (Rot) but not NaN3 or antimycin A (AntA) blocked the induction of mitochondria superoxide by adiponectin (C). Magnification, 400×.

MRC is a major source of reactive oxygen species (ROS), which stimulate UCP2 expression [33], [34], [35]. By applying a fluorescent probe for mitochondrial superoxide, MitoSOX, it was found that portal vein injection of adiponectin induced a transient superoxide burst (Figure 7A). This was concomitantly associated with the elevation of UCP2 expression and the recruitment of hnRNP K into mitochondrial fractions as shown in Figure 2 and 5, respectively. Note that the positive MitoSOX staining signal was found to be either co-localized with or close to that of von Willebrand factor (VWF), a marker for endothelial cells (Figure 7B). Flow cytometric analysis further confirmed that adiponectin could induce a short burst of mitochondrial superoxide production in HUVEC, which was detected at 30 minutes of treatment. The elevation of ROS was no longer detectable after 1.5 hours (Figure 7C). Adiponectin could not promote the transient induction of mitochondrial superoxide in H4IIE hepatoma cells (data not shown). Treatment with rotenone, but not antimycin A and NaN3, blocked adiponectin-induced mitochondrial superoxide production in HUVEC (Figure 7D). Inhibition of the NADPH oxidase by diphenylene iodonium (0.5 µM) or apocynin (1 mM) had no significant effect on adiponectin-evoked ROS generation and UCP2 expression (data not shown).

## Discussion

Uncoupling protein 2 (UCP2) is a mitochondrial inner membrane carrier protein encoded by nuclear genome [30]. The first member of uncoupling protein family, UCP1, was discovered as a physiologic mediator of proton leak and cold-adapted thermogenesis in brown adipose tissue [36]. UCP2 protein shares 59% amino acid identity to that of UCP1 [37]. However, the biological functions of UCP2 remain poorly defined. Nevertheless, a growing body of evidence suggests that UCP2 plays a beneficial role in various stages of fatty liver diseases and could exert anti-steatotic and anti-inflammatory activities through promoting mitochondrial respiration, attenuating non-esterified mitochondrial fatty acid accumulation and ROS production, inhibiting inflammatory cytokine expression and activating AMP-activated protein kinase [30], [31], [33], [35]. In mice, absence of UCP2 is associated with elevated production of ROS, increased oxidative stress, and delayed liver regeneration [33], [34], [35], [38], [39]. On the contrary, there lacks sufficient information on whether replacement with UCP2 could alleviate NAFLD [30]. In fatty liver tissues, the lipid-laden hepatocytes express high levels of UCP2, whereas the expression of UCP2 in Kupffer cells decreases [40]. The present study demonstrates that UCP2 expression is significantly reduced in NPCs, but not PCs fractions of AKO livers. Moreover, administration with adiponectin selectively promotes UCP2 expression in hepatic endothelial cells. These evidence suggest that a cell-specific regulation of UCP2 may be crucial for adiponectin to elicit its hepatoprotective functions.

**Figure 8:**
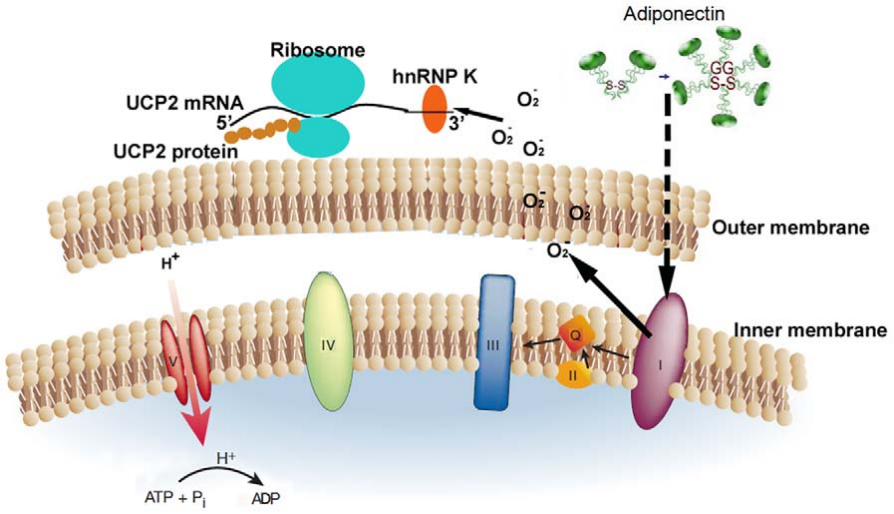
A schematic summary: Adiponectin-evoked transient elevation of mitochondrial O_2•_^−^ serves as a trigger for the translocation hnRNP K, which subsequently promotes the stabilization of UCP2 mRNA and its protein synthesis in mitochondria of hepatic endothelial cells.

The mitochondrial superoxide anion radicals (O_2•_^−^) are generated as the by-products of oxidative phosphorylation, from electrons leakage at complex I and III of the electron transport chain [41]. When tightly controlled, mitochondrial ROS act as signalling messengers for many biological responses, including the antioxidant mechanisms for restoring cellular redox homeostasis [42], [43], [44]. Here, the results demonstrate that adiponectin treatment induces the generation of mitochondrial superoxide, which can be inhibited by rotenone, the complex I inhibitor but not by antimycin A, the complex III inhibitor. Complex I (H^+^-pumping NADH:quinone oxidoreductase) plays an important role not only in cell respiration, but also in cellular/organismal ROS homeostasis [45]. Complex I-derived O_2•_^−^ production due to reverse electron transport can be inhibited by rotenone and is sensitive to the pH gradient across the mitochondrial inner membrane [46], [47]. The high rate of superoxide production by complex I could be abolished by uncoupler [48], [49]. However, the mechanisms of superoxide production by complex I are less clear than that of complex III. Nevertheless, the results of the present study suggest that adiponectin preferentially acts through a rotenone-sensitive and complex I-related pathway to induce the superoxide burst and the expressions of UCP2 (Figure 8). Elevated UCP2 subsequently maintains low ROS production in the mitochondrial matrix [38].

Both adenovirus-mediated chronic overexpression of adiponectin and acute injection of the recombinant protein elevate mRNA and protein levels of UCP2 in mitochondria. Transcription inhibitor actinomycin D does not affect the ability of adiponectin to stimulate UCP2 expression. Adiponectin stabilizes and facilitate the transportation of UCP2 transcripts to mitochondria-associated polysomes for protein synthesis, which involves hnRNP K, a nucleic acid binding protein. Different hnRNP proteins affect distinct sets of targets. hnRNP K is one of those showing the broadest effects on regulating mRNA transcription, stability and translation of genes involved in the control of apoptosis, oxidative stress, oncogenesis and metabolism [50]. A large number of RNAs associated with hnRNP K are nuclear-encoded but expressed in mitochondria, including UCP2 [51]. Translocation of hnRNP K from nuclear to cytoplasm and the importation of this protein into mitochondria have also been implicated in regulating mitochondrial genome transcription [52]. hnRNP K binds with the 3′-untranslated region and increases the abundance of UCP2 mRNA in mitochondria [32]. Mitochondria content of hnRNP K in AKO livers is much lower than that of C57 mice. Similar to UCP2 elevation, adiponectin-induced mitochondrial translocation of hnRNP K can also be inhibited by rotenone treatment. These evidence indicate that adiponectin-induced hnRNP K translocation to mitochondria could be a downstream response to the transient mitochondria superoxide production (Figure 8). Indeed, previous studies have suggested that a number of redox-linked signal transduction pathways, such as protein kinase C [53], c-Jun N-terminal kinases [54], c-Src [55], and extracellular signal regulated kinases [56], are involved in the activation of hnRNP K.

In summary, the present study demonstrates that adiponectin treatment in liver induces a transient superoxide production from mitochondria, which initiates a signalling event for UCP2 up-regulation through hnRNP K (Figure 8). Based on experiments with pharmacologic inhibitors, complex I inhibition (with rotenone) but not complex IIII inhibition (with antimycin A) decreases the superoxide generation induced by adiponectin. To characterize the specific sites of superoxide generation and the downstream ROS signals may be attractive targets for the development of adiponectin-based hepatoprotective therapies.

## Materials and Methods

### Materials

Sodium azide (NaN_3_), rotenone (Rot), antimycin A (AntA), cycloheximide (CHX) and antinomycin D (ActD) were purchased from Sigma Aldrich (St. Louis, MO, USA). Dulbecco's modified Eagle's medium (DMEM) and mito-hydroethidine [MitoSox, 3,8-phenanthridine diamine, 5-(6′-triphenyl phosphonium hexyl)-5,6 dihydro-6-phenyl] were purchased from Molecular Probes (Invitrogen, Carlsbad, CA). Bicinchoninic acid (BCA) protein quantitation kit was purchased from Pierce Biotechnology Inc. (Rockford, IL, USA). Polyvinylidene fluoride (PVDF) membrane and chemiluminescence solution were obtained from GE Healthcare (Piscataway, NJ, USA). Unless otherwise stated, all chemicals were purchased from USB Corporation (Cleveland, Ohio, USA). Rat hepatoma cell H4IIE, human umbilical vein endothelial cell (HUVEC) and mouse marcrophage RAW264.7 were obtained from ATCC (Manassas, VA, USA). Human stellate cell LX2 was a kind gift from Dr George K. K. Lau (Clinical Trial Center, the University of Hong Kong). Cells were cultured with DMEM containing 10% FBS, 100 U/ml penicillin and 100 µg/ml streptomycin at 37°C in a humidified atmosphere of 5% CO_2_. Murine adiponectin recombinant protein was purified as described [57].

### Animal studies

C57BL/6J mice were purchased from the Jackson Laboratory (Bar Harbor, ME, USA). AKO mice in C57BL/6 background was a kind gift from Prof. Lawrence Chan (Baylor College of Medicine, Houston,TX, USA) [24]. All animals were kept under 12-hour light-dark cycles at 22–24°C. The mice were fed with standard chow containing 20% protein, 10% fat and 70% carbohydrates (LabDiet 5053, Purina mills. Richmond, IN). Male mice at the age of 6–8 weeks were used for this study. Adenovirus expressing luciferase or adiponectin was administered into mice by tail vein injection at the optimal dosage of 1×10^8^ pfu per mouse [24], [31]. For drug treatment, actinomycin D (5 µg/ml), cycloheximide (40 µg/ml), rotenone (20 µM), NaN_3_ (2% w/v) or antimycin A (20 µM) in 100 µl PBS were injected into portal vein with or without adiponectin. All animal experimental procedures were approved by the Committee on the Use of Live Animals for Teaching and Research, the University of Hong Kong (No 1655-08), and carried out in accordance with the Guide for the Care and Use of Laboratory Animals published by the US National Institutes of Health (NIH publication 86-23 revised 1985).

### Isolation of PCs and NPCs from liver tissues

Mice were sacrificed under deep anaesthesia. Liver perfusion was performed as described [58]. After removing cellular aggregates and tissue debris, cell suspensions were filtered through a cell strainer (mesh width 100 µm). PCs were enriched by low speed centrifugation (500 g) for three minutes at 4°C. The NPCs supernatant was saved for further fractionation using a two-step Percoll gradient (50% at the bottom and 25% in the upper layer). After centrifuging at 800*g* for 15 minutes at 4°C, endothelial cells-enriched fractions were collected from the intermediate zone between the two Percoll layers. Fractions containing Kupffer cells were obtained from the 50% Percoll layer. Mitochondria were isolated from total liver lysates, PCs and NPCs by following the protocol described previously [24].

### In vivo and in vitro detection of mitochondrial reactive oxygen species (ROS)

Mitochondrial superoxide was detected by MitoSOX RED™, a fluorescent probe targeted to mitochondria. MitoSOX exhibits fluorescence after oxidation by superoxide (excitation 510 nm, emission 580 nm). For *in vivo* evaluation, MitoSOX with or without adiponectin (50 µg per mouse) in a total volume of 100 µl was administered into portal vein. The liver tissues were collected at different time points for preparing the 5 µm cryo-sections. After fixation in ice-cold acetone and counter-stained with DAPI, images were captured with Olympus BX41 microscope (Olympus, Tokyo, Japan). von Willebrand factor (VWF) was used as an endothelial cell marker for immuno-staining in the liver tissue sections. For determination of mitochondrial superoxide by flow cytometry, HUVEC were preloaded with 5 µM MitoSOX for 30 min in medium without phenol red. After washing, adiponectin with or without various inhibitors were added into the cells for different periods prior to flow cytometric measurement [59]. Triplicate experiments were carried out for each set. The data were presented by histogram of mean intensity of MitoSOX fluorescence. The analysis was performed on the Cytomics FC 500 (Becman-Coulter, CA, USA).

### Real time quantitative polymerase chain reaction (QPCR)

The cDNA reversely transcripted from total RNA was used to analyze gene expression levels. Fluorescent SYBR Green (Qiagen, Hilden,Germany) was used to quantify the PCR product on an Applied Biosystems Prism 7000 sequence detection system. The primer sequences were: Forward 5′-GGATACTGCTAAAGTCCGGT-3′ and Reverse 5′-CCATTGTAGAGGCTTCGGG-3′ for *UCP2* (NM_003355.2); Forward 5′-TAGTGCGCACCGCAGCC-3′ and Reverse 5′-AGCTCATCTGGCGCTGCAG-3′ for mouse *Ucp2* (NM_011671.4); Forward 5′-GAGAGTCAAGGGCTAGCGC-3′ and Reverse 5′-GCTTCGACAGTGCTCTGGTA-3′ for rat *Ucp2* (NM_001130033.1); Forward 5′-TGACCCAGATCATGTTTGAGA-3′ and Reverse 5′-AGTCCATCACGATGCCAGT-3′ for human *ACTB* (NM_001101.3); Forward 5′-AGTGTGACGTTGACATCCGT-3′ and Reverse 5′- CCACCGATCCACACAGAGTA-3′ for mouse *Actb* (NM_007393.3); Forward 5′-CACCCGCGAGTACAACCTTC-3′ and Reverse 5′-CCCATACCCACCATCACACC-3′ for rat *Actb* (NM_031144.2); Forward 5′-ACGAAATCAACAACCCCGTA-3′ and Reverse 5′-GGCAGAACGACTCGGTTATC-3′ for mouse *MT-Co2* (NC_005089.1). β-actin gene (*ACTB* or *Actb*) was used as the QPCR internal control for cell and tissue RNA samples. Cytochrome C oxidase II (COXII) gene (*MT-Co2*) was used as the internal control for mitochondrial polysome-associated RNA samples. The amplified products were checked by separation on a 2% agarose gel.

### Western blotting

Tissues were homogenised in lysis buffer [20 mM Tris-HCl, pH 7.5, 150 mM NaCl, 1 mM Na_2_EDTA, 1 mM EGTA, 1% Nonidet P 40, 0.1% (w/v) SDS, 1% (w/v) sodium deoxycholate, 1 mM NaF, 1 mM Na_3_VO_4_, 1% (v/v) protease inhibitor cocktails (Roche, Mannheim, Germany)]. Protein lysates were heated at 95°C for 5 min, separated by SDS-PAGE, and transferred to PVDF membrane for immunoblotting with the specific antibodies against UCP2 (R&D Systems, #AF4739), single-strand binding protein-1 (SSBP-1, Santa Cruz, #sc-34727), liver sinusoidal endothelial cell marker SE-1 (Novus Biologicals, NB110-68095), F4/80 (Abcam, #ab6640), hnRNP K (Santa Cruz, #sc-25373) or β-actin (Sigma, #A5316). Immune-complexes were visualized using enhanced chemilumincesence. SSBP-1 and β-actin were used as loading controls for mitochondrial and total cell lysates, respectively.

### Statistical analysis

All results were derived from at least three sets of experiments. The density of protein bands in Western blotting results were quantitatively analyzed by ImageJ software for calculating the expression ratios against loading control. Representative Western blotting images were shown. Values are expressed as mean ± SEM. The statistical calculations were performed with the Statistical Package for the Social Sciences version 11.5 software package (SPSS, Inc., Chicago, IL). Differences between groups were determined by Student's t-test. Comparisons with *P*<0.05 were considered as statistically significant.
